# The Use of Porcelain in Dentistry

**Published:** 1909-02-15

**Authors:** Chalmers J. Lyons

**Affiliations:** Jackson, Michigan


### THE USE OF PORCELAIN IN DENTISTRY.

BY CHALMERS J. LYONS, D.D.S., JACKSON, MICHIGAN.

Read before the Michigan First District Dental Society, Dec. 10, 1908.

The subject which your committee has asked me to discuss to-night is "The Use of Porcelain in Dentistry."

It is not without a feeling of considerable embarrassment that I attempt to impress my feeble efforts upon this body, when I see men around me, men who are better qualified to discuss this subject than am I. If, however, some interesting points can be brought out in the discussion of this paper, then I shall feel that my efforts have not been wholly in vain.

Two years ago an entirely different paper on the Uses of Porcelain in Dentistry might have been written, for within this short time the writer feels that the field of porcelain in dentistry has been narrowed down to an extent that has been quite perceptible to one who is daily manipulating it in crown and inlay work. While the field has been narrowed down in crown work by the process of casting in gold the abutments for crowns, and narrowed down in inlay work by the cast gold inlay, and perhaps also by the silicate cements, there still remains a broad field for porcelain that can not be occupied by any of the other materials we now have for the purpose of restoring lost natural tooth structure.

The extent to which the silicate cements have cut in on the field of porcelain, clinical experience is at this time, too limited to justify a definite statement. However, if they prove worthy of being classed among the permanent filling materials, no doubt they will at least be indicated in the small approximal cavities in the anterior teeth, but in the large restorations it is extremely doubtful if they will ever supersede porcelain on account of the opacity of the material.

The successful employment of porcelain in dentistry is dependent on a definite knowledge of the fundamental principles and requirements, and upon the acquirement and development of skill, together with a thorough knowledge of the nature and properties of the material. It is the lack of this knowledge, so essential to the successful application of the material that has caused many failures and subsequent condemnations of porcelain. Dr. Miller has summed up the qualities which the ideal filling material should possess, as follows:

(1) Sufficient strength: that it may neither break nor wear away under the stress of mastication.

(2) Chemical indestructibility: that it may remain unaffected by the fluids of the mouth, or by food or drink.

(3) Permanence of form and volume in the mouth.

(4) Thermal non-conductivity: that changes of temperature in the mouth may not be conveyed to the pulp.

(5) A high degree of adaptability: that it may be made to fit the walls of the cavity so closely as to exclude moisture.

(6) Color resembling as near as possible the color of the tooth to be filled.

(7) Absence of every quality injurious to the substance of the tooth, including the pulp, or to the mucous membrane or to the general health of the patient.

(8) Ease of insertion.

(9) The least susceptibility to moisture.

The old maxim, "Aim at the highest prize and if there thou fail, thou will surely reach one not far below," is a good one to live by in any of the walks of life and it should be ever in the mind of the conscientious dentist. In every dental operation the highest ideal should be sought for. In performing an operation which is ideal, a material which is as near ideal as it is possible to secure, must be brought into practical application with the highest development of skill and a thorough knowledge of its nature and properties.

It is now pretty generally conceded that the cemented filling makes the nearest approach to the ideal in the large restorations, because the cemented fillings make an absolutely water-tight filling. This I believe to be one reason that recurrent decay is so seldom found around an inlay. The position of the teeth in the mouth, the quality of the tooth structure, and the size of the cavity of decay, are the conditions upon which the adaptability of porcelain fillings depend.

Before the advent of the cast gold inlay, porcelain as a filling material was indicated in many places, which now can be more successfully treated by means of that process.

Because of the friability of the material, porcelain is not indicated in the large occlusal or occluso proximal cavities in bicuspids and molars, except it be for cosmetic effect, for the cast gold inlay in these large cavities has all of the advantages of the porcelain inlay, together with sufficient strength that it will neither break nor wear away under the stress of mastication.

For the large restorations in the anterior teeth the porcelain inlay is very nearly the ideal filling, and when we consider one by one Dr. Miller's requirements of an ideal filling material, we shall find that porcelain meets most of these demands for this class of cavities.

In order to make this ideal filling material into an ideal filling, a most thorough knowledge of the nature of the material must be had that it may be properly fused in order to bring out the required strength and to retain its original color.

Porcelain is a material composed of silicon oxide, the silicate of aluminum, potassium and sodium which becomes a hard, dense mass by the process of fusion. Unless the operation of fusing is successful, the hard dense mass is not procured. Dr. John Q. Byram, in the *Items of Interest*, says: "The baking or fusing of the porcelain body is one of the most important factors in porcelain, as the results as a whole depend upon this successful performance."

Density, strength, shade and surface gloss are effected by and dependent on the proper conduct of fusing.

Too rapid heating or over fusing will effect the density and strength by causing porosity and brittleness, and insufficient fusing will impair its crushing strength and gloss.

The given shade of any porcelain depends on its fusing at exactly the heat intended for that special preparation.

The glaze is defective if insufficient heat has been applied and a glass like appearance is imparted to the surface by overfusing.

Shrinkage is a property of porcelain that must be studied in order to be able to bring out the best that is in the material. While shrinkage in porcelain can not be overcome on account of the nature of the material, it can be controlled so that the operator may definitely know how much shrinkage to prepare for in making a crown or inlay.

This can be accomplished by having each mixture of the porcelain body of a uniform consistency, mixing it just as stiff and dry as is possible to manipulate it and by very thorough condensation drive to the surface and absorb the surplus moisture until the piece is hard and firm. By so doing the crown or inlay will take on the minimum amount of shrinkage in fusing, and it will be more homogeneous in texture.

Uncleanliness is frequently responsible for failure in procuring density to the piece, as any foreign matter in the porcelain body or on the platinum upon which the porcelain body is to be placed for fusing, will be turned into gases in the process of fusing, and these gases in attempting to escape will cause porosity in the crown or inlay.

The hardest problem to contend with in the use of porcelain is to obtain the proper shades of color, and as the cosmetic value is the chief factor in the indication of porcelain in dentistry, in the use of this material a keen eye for color must be developed, and a most thorough knowledge of color combinations and the influence which effect them must be understood.

In order to comprehend and apply the principles of colors in porcelain work the foundation principles of light has to be reckoned with.

The relation of color to light is much the same as that of music to sound. Color has its many hues, its long scales of tints and shade, its true and false chords.

Mere sound gives us little pleasure, when developed, however, into its highest form, music, we are thrilled.

So in light our enjoyment culminates at the glories of color in a flower or in a sunset or in some of nature's paintings. The harmonies of light have not been as thoroughly studied and classified by scientists as have those of sound. If they had been, the porcelain operator would have less difficulty in imitating the tones of color in the natural teeth.

The ability to imitate the various hues and tones of color of a tooth depends as much upon our susceptibility to the various gradations of color as upon our skill in the manipulation of the materials.

It is a well known fact that there are some individuals with such perfect organs of hearing that they are able to distinguish the slightest sound who yet are so utterly unable to distinguish between two tones or between harmonies and discords of music that they are said to have "no ear." So there are those whose eyes are as well formed for seeing all and distant objects, but who are unable to distinguish between the hues and tones of color and may be said to have "no eye for color."

To successfully comprehend and apply colors in porcelain one must have an eye for color. Then the eye can be developed to distinguish the finer hues and tones only by close application to the work and by observation and scientific study of colors.

The operator need not fail in obtaining the proper colors on account of the chemical change taking place in the pigments during the fusing of the porcelain. There is more danger of failing to bring out the proper tone of color in the process of fusing of porcelain by not obtaining the proper surface gloss, than there is in chemical decomposition of the pigments taking place.

If the surface gloss of porcelain is not the same as that of the natural tooth than the reflection of light will be different and the appearance changed. If the inlay is underfused there will be lacking the required amount of reflection of light, if overfused, and a gloss-like surface produced, the reflection of light will be too great and the inlay will not match the tooth in tone of color, even though all the pigments of color are preserved in normal condition in the porcelain and in the right relation for that particular case.

In building up the porcelain body to form the inlay, doubtless the layer method is the best for the operator who has had long experience and who has made the study of color and color combinations a part of his daily work, but to the man with a limited experience in color combining, it is too indefinite for practical application. Every layer of body in succession is influenced by the underlying layers and the degree of modification in color is principally dependent upon the thickness of each successive layer, the degree of translucency hue and tone of color and intensity of colors. These are all guides to the final results to one who has had long experience in combining colors, either in chromatic art, or in ceramic art where their experience may signify to them just what tone of color that a definite enamel color applied in a definite thickness of layer will bring out by applying it in a certain position in the inlay over another layer already applied and partially fused.

To the novice or porcelain operator with a limited experience these things are necessarily indefinite and not positive, and to them in building up in layers, the thickness of the layer, and intensity of tone of color of that layer is necessarily guess work to a greater or lesser degree, and the final results are many times disagreeably surprising..

To the operator who has not had the opportunity to make a scientific study of color and color combining, the method of building up the inlay in sections will be more positive and more satisfactory. This is accomplished by building in with the foundation body that part of the inlay which is to represent the dentine and fusing to a high biscuit, then build in the gingival third of the inlay with tones of color of the gingival third of the tooth, then the middle third, then the incisal third, not having the layers overlap each other. The inlay is completed with a uniform color. While in a measure this may be considered a layer method, yet the enamel layers do not overlap, and the tone of color is not influenced by an enamel layer underlying.

Under different sources of light by which an inlay is illuminated, it will appear differently regarding the color, and it is possible to produce an inlay which will exactly imitate the hues and tones of color of the natural tooth only under certain definite conditions. The position of the inlay in the tooth with reference to the source of light must always be taken into consideration before attempting to build up the inlay with colors.

An inlay on the distal surface of a tooth should always be made lighter in tone of color than one on the mesial, for the one on the mesial surface receives the rays of light with less obstruction than the one on the distal surface.

If an inlay be so constructed that the light can not penetrate through, unchanged to the cement, the color will not be nearly so greatly modified when an inlay is cemented to place.

The dentine of a natural tooth under a high power glass has the appearance of having a rough surface. The nearer that rough surface can be imitated with the foundation body which is to represent the dentine, the less will the color of the inlay be modified by the cement. The reason of this is that the rays of light are broken up at the junction of the foundation body and enamel, or, at the rough surface, and instead of the rays of light passing straight through the cement, we get diffusion of light and instead of all the light being refracted, reflected and absorbed, a part of it at least, is diffused the same as we have in the normal natural tooth.

This rough surface on the foundation body can be accomplished in one of two ways: eithe by using consolidated body for the foundation fixing to a high biscuit, and not any time thereafter fusing, and in using Brewster or S. S. W. body for enamel colors, or it can be left rough by roughing the surface after the surplus water has been absorbed, then by fusing to a high biscuit and at no time over-fusing the foundation body in fusing the enamel body to complete the inlay. I have been able to get splendid results with these methods of procedure in applying the foundation body, and I firmly believe that the nearer we can imitate the appearance of the normal dentine with the foundation body, the nearer we can come to procuring the color of the natural tooth with our inlays, and that the porcelain inlay will be nearest the ideal filling for the large restorations in the anterior teeth of anything we now have.

The significance and comprehension of cavity preparation plays no little part in obtaining the highest ideal in the use of porcelain as a filling material and cavities, for porcelain inlays must be so formed as to not only retain the inlay but must be shaped in such a manner that the inlay will have sufficient strength when the inlay is set in position. Disregarding the foundation work or principles of cavity preparation for inlays, many dentists have become discouraged because they began to construct inlays before they had studied these principles.

The preparation of many cavities for inlays requires the sacrifice of sound tooth structure in order to secure sufficient strength and the retentive resistance that is necessary, and to obtain proper color effect. Sufficient strength, that it may neither break nor wear away under the stress of mastication is the first of the requirements given of an ideal filling by Dr. Miller. In order to obtain sufficient strength, we have to have sufficient bulk of material in a porcelain filling, and in order to get sufficient bulk in many inlays it is necessary to sacrifice sound tooth structure. It is necessary in many inciso proximal cavities to sacrifice sound tooth structure on the lingual surface for the purpose of forming a step in order to insure the retentive resistance that is necessary to make a permanent operation.

In many cavities, particularly on the labial surfaces, it becomes necessary to sacrifice sound tooth structure to obtain proper color that the light may not penetrate through the inlay to the cement unchanged. Unless the case will justify the sacrifice of enough tooth structure to obtain these requirements, then the porcelain inlay is not indicated.

The principles of retention in the preparation of the cavities for inlays and for fillings are widely different. The preparation of a cavity for an inlay requires more exactness in its details than that for a gold or amalgam filling. Unless the foundation principles of cavity preparation for inlays are firmly grounded and the wide contrast between the preparation of a cavity for a porcelain inlay and that for a filling is recognized, we can not hope to accomplish the highest degree of success in the application and retention of the porcelain inlay.

Some writers and operators advocate the retention of the porcelain inlay by means of close adaptation of the inlay to the cavity walls and a thin film of cement. This method is well and good for cavities existing where no stress is exerted on the inlay during the process of mastication such as in the cervical or buccal cavities, provided the inlay and cavity be grooved before setting the inlay, but for cavities on the occlusal proximal or inciso proximal surfaces, I believe that not only do we have to have close adaptation and frictional retention, but in addition thereto, it is necessary to have mechanical retention if the inlay is expected to take its place with the permanent filling materials, that is, grooves and angles should be made in the cavity that the inlay when completed will be so shaped that in a measure it will be self-locking in the cavity.

The subject of cavity preparation for inlays is too broad for the time allotted me to-night to take it up in all of its details, so I will only take the time to mention a few of the essential requirements.

1st. The force and direction of occlusion must be taken into consideration before beginning the preparation of the cavity, then by grooves and angles secure all the mechanical retention that is possible without forming undercuts.

2nd. The walls of the cavity should slightly diverge toward the margins.

3rd. The cavity should be as deep as conditions will permit with the pulpal wall parallel to the plane of the surface on which the cavity is located.

4th. All undercuts must be obliterated so as to be able to withdraw the matrix without destorting it.

5th. Frictional retention must be secured by having the pulpal wall as extensive as possible without forming undercuts.

6th. Sufficient working space must be secured before beginning the operation.

If these essential laws are not carried out, nothing can be expected in porcelain inlay work except failure, and if the conditions presented will not justify following out every one of these principles then, in my opinion, some other material would be indicated for the filling.

What has been said regarding the nature of the material, the acquirement and development of skill and the training of the eye to detect the different hues and tones of color, is as essential in the use of porcelain work in crown or bridge as in inlay work.

There is no material we now have for executing crown work which so nearly approaches the ideal as does porcelain, one which so nearly imitates the conditions of nature not only from a cosmetic standpoint but from a physiological standpoint as well, for it possesses no quality which is injurious to the mucous membrane or to the general health of the patient.

Porcelain is a mineral substance and possesses the characteristics of friability and as strength is a requirement of equal importance with the esthetic in crown work, this must be taken into account in using this material in the construction of a crown. The use of porcelain in crown work in its present form with the facilities available, makes it possible for the experienced and skillful operator to achieve results which combine both the requirements of beauty and strength.

In view of the friable nature of the material the necessary degree of strength is not obtained only by the presence of a sufficient thickness or bulk of material to insure an adequate degree of resistance to stress during the process of mastication.

While failures will occur in any line of work, they need not occur in porcelain crown work, on account of the friability of the material, if the operator is judicious in the application and has a clear conception of the structural requirements.

In porcelain crown work the metallic structure constitutes the foundation and it should be made just as strong and rigid as it is possible to make it as the strength of the piece is dependent to a great degree upon the foundation.

The line of union or the joints of the section of the metallic parts should be closely fitted so as to insure perfect contact so as to require the minimum amount of solder in uniting. Pure gold or gold and platinum solder should be used to unite the parts for gold alloyed with the baser metals would oxidize in the process of fusing the porcelain and the gases would cause porosity of the piece.

The application of porcelain to the construction of crowns is contra-indicated in all cases where a sufficient thickness of porcelain body can not be obtained on account of the close occlusion of the opposing teeth to insure an adequate degree of strength that will resist the stress of mastication. As this condition is not frequently presented except in molars, porcelain may be said to be indicated for crown work on the ten anterior teeth, and in cases where there is sufficient room between the opposing molars to obtain a sufficient bulk of porcelain, this material may be used there with good advantage.

While porcelain may be used in the ten anterior teeth principally for its aesthetic and hygienic value, it is not usually necessary from the aesthetic standpoint that it be indicated on the molars but from the hygienic standpoint it has an inestimable value for it presents a smooth highly polished surface which is more easily kept clean than gold, because it is not effected by the chemical action of the secretions of the mouth, and food products will not cling to or become deposited upon it.

Dr. Goslee in his book on the "Principles and Practice of Crowning Teeth," says: "In the application of porcelain crown construction, the highest possible advantages can be derived only from a careful observation of the requirements, combined with a skillful execution of the details in the preparation of the root, the construction and adaptation of the cap and attachment of the facing, and the manipulation of the body itself.

When the conditions of occlusion are, or may be made favorable, and when these details of construction have been executed with skill, a porcelain crown possesses adequate strength to meet the requirements of all average and typical cases, and the possible integrity in such work often exceeds that of any other style of construction."

The possible integrity of life of any crown is dependent to a great degree upon the preparation of the root to receive it. For some years it has been my practice to use the plate and dowel instead of the cap and dowel in all of my porcelain crowns on the six anterior teeth. The root is prepared by beveling the root both labially and lingually from a central point or from the root canal, so that the plate which is to form the base of the crown straddles the exposed end.

The labial bevel should extend from the center of the pulp canal to a point sufficiently far beneath the gum to allow for the thickness of the plate. The lingual bevel should extend to the gum line. As the stress of mastication is upward and outward on the six anterior teeth, the lingual bevel will act as a support for the entire root when stress is brought to bear on it, thus preventing fracture of the root.

When the root is prepared in this manner the normal physiological conditions of the gum tissues are not disturbed and in using porcelain to construct the crown, the hygienic and aesthetic advantages are almost ideal in replacing a lost natural tooth by an artificial substitute.

In constructing the porcelain crown, after the desired root preparation has been secured a piece of platinum, gage about 36, should be cut a trifle larger than necessary, and annealed and burnished to a perfect adaptation with the surface of the root. After securing the proper adaptation of the plate, the canal should be prepared for the reception of the dowel which should be of iridio platinum, a trifle longer than the length of the crown when completed. The plate and dowel should now be united with pure gold or gold and platinum solder and plate again reburnished to the root.

A Consolidated facing of the desired mould and shade is now placed in position and soldered to the dowel with pure gold and platinum solder, and when united, the solder evaporated by means of high heat either with an oxyhydrogen blow pipe or by means of the electric furnace. In placing the facing in position a space of about one milemeter in width should be left between the plates and the facing in order to permit of reburnishing the platinum after the first fusing of the porcelain body.

In applying the porcelain body on the lingual surface care must be taken not to build over the lingual plate until after the first fusing of the porcelain body and the reburnishing of the platinum, as the shrinkage of the porcelain will have a tendency to draw the platinum away from its original adaptation.

When using the Consolidated facing, Consolidated porcelain body should be used for when the final fusing is completed the crown will be one homogeneous mass, and will be much stronger than when the facing and the porcelain body have different fusing points.

In the construction of the bicuspid porcelain crown, I use a half band or a band on the lingual half. The stress in mastication in these teeth is upward and laterally and the half band supports the root in the lateral stress, the half band being made of iridio platinum. The principles of constructing these crowns are much the same as for the anterior crowns only on account of there being no physical union between porcelain and platinum; it is not only desirable to make as much space for the porcelain which is to form the entire lingual surface of the crown as possible, but it is necessary to provide some means of supporting it against any possible line of cleavage in order to preclude subsequent fracturing from the stress of mastication. This may be accomplished by attaching a small, round platinum pin to the center of the lingual portion of the cap, or by using the surplus end of the lingual dowel when two dowels are used. Care must always be used in obtaining the minimum amount of metal in the construction of a porcelain crown for it is the bulk of the porcelain body that has to be depended upon to furnish the adequate strength.

Molar crowns in porcelain may be made in a similar manner to the bicuspid or the entire crown may be built up with porcelain body without a facing.

Dr. F. W. Howlett, of Jackson, Michigan, has recently brought out a method for articulating porcelain whereby one may get an absolutely perfect articulation by its use in building up molar crowns. The method is rather a long one and the time is too limited to-night to describe it in all of its details, so I will only give an outline of it. It consists in taking a bite in Stents compound, a very hard modeling compound, and after carving up the cusp a matrix is made of the occluding surface in which porcelain is fused to form the cusp of the crown. A porcelain base is built on the abutment of the crown, and then the porcelain cusp is set on the base in the right relation with the opposing teeth and waxed in position with a small amount of casting wax. Porcelain body is then applied between the cusp and base and fused. The crown being completed by several subsequent bakings.

Porcelain is surely indicated in the restoration of the peg laterals by means of the all-porcelain jacket crown. This, no doubt, represents the highest achievement in porcelain work, and if it had no other use in dentistry it would be a material which would receive the benedictions of many thankful people.

The use of porcelain in bridge work I believe has a limited field on account of the friable nature of the material.

It is only indicated in those cases where there has been a great amount of absorption, and where there is a good opportunity to get a large bulk of material and is probably indicated for removable bridge work more than for fixed bridges and particularly so in large bridges.

In the construction of a porcelain bridge the foundation or frame work necessarily has to be made very strong, and in order to get strength a large amount of metal has to be used which would detract from the strength which might be imparted to it by the porcelain. On account of the hygienic advantages of porcelain, is is lamentable that its mechanical advantages are not such that will justify its more practical application in bridge work.

The use of porcelain in the continuous gum work, no doubt, represents the highest achievement in prosthetic dentistry. From a hygienic standpoint it stands without a peer. However, it has its disadvantages in the fact that it requires a manipulative skill which can only be obtained by long experience and a thorough knowledge of the details in that particular line of work, and which the large majority of operators do not have the opportunity to procure.

In conclusion let me say that in the practice of dentistry we should never let our enthusiasm over-rule our better judgment, we should not be extremists in any one branch. Porcelain has a field which at the present time can not be occupied by any other material. Gold has its place and on account of its durability will ever stand in the first ranks as a filling material and also as a material for the construction of crowns and bridges. Let us combine these two great materials in our practice and when the hygienic and aesthetic requirements predominate let us use porcelain, but when the requirement of strength is a predominant feature let us not hesitate to use gold, thereby uniting all that is best in the two materials and rendering to our patients the best possible service at our command.

### DISCUSSION.

DR. J. M. THOMPSON: To those of us who attended the state meeting in June and witnessed the clinic given by the members of the Second District Dental Society, this paper does not come as a surprise, because I am sure that all admit that Dr. Lyons has over-stated the fact a little bit when he said that, "we, in Detroit, set the pace." I think that the members on that day set a pretty rapid pace for the First District, in the use of porcelain especially. In view of the statement he made in his paper that it is harder to write a paper to-day on the use of porcelain than it was two years ago, I have wondered what we would have done two years ago if he had had an opportunity.

A recent experience with the enamels, such as Ascher's, Translux, and so on, has led me to make this request: That we confine ourselves to the discnssion of porcelain to-night, and not these cements. I hope these may be taken up by us later on, as they are worthy of discussion. Dr. Lyons outlines the very nice comparison between the art of selecting colors and the art of music. Now, if it was as easy for us to select colors as it is for one to go to a piano and strike a given chord or different tone, the selecting of colors would simply be a matter of practice.

We take the porcelains that are given us and are designated by different shades, and we mix them together, and we expect certain results, and perhaps some of our results are as unpleasant to us as a discordant violin being played by a six-year old in the next flat. The different filling qualifications, as outlined by Dr. Miller, have probably set a standard for all, and it is no doubt owing to the friability of porcelain that it has fallen somewhat into disuse and the gold inlay has taken its place to quite an extent. Dr. Reeves said, and I think I have heard Dr. Raymond make the same statement on paper, and I think I have made the same myself on one or two occasions, that the use of porcelain was limited only by the skill of the operator. I still believe it. Think it over.

There is no question that the fusing of porcelain is one of the most important points in the use of this material. It is to be lamented that we have not at the present time facings made purposely for porcelain crown work, that is, a facing that is a little under-done and of a given color. Facings, partly fused, would greatly aid us in the results that we seek to obtain, because in the use of the "Consolidated" porcelain, as Dr. Lyons has said, the crown becomes a homogeneous mass, and there is great danger of over-fusing, especially if grinding has been done upon the facing to give it some special shape required for an individual case. However, that is not of so great importance as it would be if the material was of a poorer quality. I have often said that this particular material was almost "fool proof" and that you could not spoil it, but you can.

The bubbles that Dr. Lyons mentioned are very often apparent when the utmost care has been exercised.

The point he brings out about foundation body and the use of it are excellent points indeed. I have found great satisfaction in using a white body furnished by the Consolidated Company. I took the idea from the white furnished by Brewster, which is of very little use where he had hoped it would be, in labial cavities, on account of its extreme whiteness and the thinness of the amount of material we use for such inlays. With this white foundation body I believe that the shadow problem is as well overcome as with any other material that we can use. In the lower fusing materials such as Jenkins' and Brewster's, almost all these materials are fluxed with glass. Now, the presence of flint glass in any porcelain is a detriment to it, on account of the percentage of lead that is in all glass preparations, and in over-fusing this lead becomes formed in little globules in the porcelain, and if it is near the surface it is dissolved or washed out and we have a porous or roughened condition of the porcelain which is so unsightly in many pieces of work.

Dr. Lyons speaks of the dowel and cap. The use of 36 gauge as a cap and the iridio-platinum dowel, is one of the oldest crown foundations we have ever used in making the porcelain crown. That is the way Dr. Downey adopted his facings which he then filled in with the Downey body. Some very creditable work was done with that material.

I believe that the time has come when operative dentistry will be subservient to the demands of oral prophylaxis, and in that respect I believe that we are going to do away with a great many root bands. As far as the band is concerned in its application to bicuspids, I think it is practically of little use, because these crowns are made just as strong without it.

With the post set in the root, and thoroughly anchored with cement, we have an opportunity to see how much porcelain we are going to have between the post and the opposing tooth. If we make the cap and the tooth fit over the post, or adjust our facing, and then build in at the back, giving ourselves plenty of room on the lingual surface, I think we have all the strength necessary for a strong and useful bicuspid crown. In fact, I do not know that I have ever had but one split, and that was done before it was set.

As I have progressed in the use of porcelain in crown work, I am gradually getting to the exclusive use of a jacket stump root formation and the making of a platinum matrix, making the jacket crown as usual and removing the platinum, my adaptations, I am sure, they are better than with a 36 gauge platinum left in the crown. In using the 36 gauge platinum, Dr. Lyons says let it extend a little over the root. It is absolutely necessary that we bring the porcelain up to the outlines of the root itself, and in many cases you will find that if you leave this platinum on and hanging over the edge of the root you will have some irritation, and the gum will move away from it in a few months. It is almost impossible to remove 36 or 40 gauge platinum from the crown after having the work completed, and have your crown fit as well as it would if you left it right in place.

It is just as easy to set the post in the root as to make it in the crown, because it is well proven that in the proportion as you bake metal into porcelain you weaken it just that much, and as far as putting in retentions on the lingual surface of the cap to increase the strength is concerned, while it makes a better union to the cap, I do not think it adds to the strength.

Dr. H. C. Raymond: The point the essayist makes about fusing is especially a good one. I think much porcelain work spoiled by improper fusing—by that I mean that the colors are lost. If it is unglazed, its refractive power is lost, and if it is over-glazed, it does not look natural with the labial teeth. One point usually lost sight of is that there is quite a difference in the gloss of the enamel of different teeth. The enamel of some teeth has little glaze compared with that of others, and that fact should be noted where work is being done.

I am a strong advocate of the layer method, because I find I can get better results from that than from mixing colors together. In that way you can fuse one or more colors together, a gentle tap brings them together, and you get a harmonious blending that you cannot get by mixing them any other way. A filling which is made of porcelain put together in that way has more of a china-like appearance. Of course, those who have not tried it think it is very difficult, but it is not so difficult as you might imagine.

Quite frequently I am asked if my fillings stay in, and many seem to have difficulty with that problem still. I think this is largely caused by some teachings of our earlier writers of five or six years ago, who created the impression that really all that was necessary was to cement the inlay into the cavity, paying no attention to retentive cavity formation. You must have a properly formed retentive cavity. I think many fillings come out because the patient is merely asked to close the mouth in direct occlusion, and are not asked to go through the motions of mastication, which would indicate adverse contact.

For a time I used nothing but a Consolidated body for my crowns, but I do not now think it is necessary. In the first place, when you are running some of these bodies through the furnace, 2,550 degrees all the time, you are going to put your furnace out of business. I find that the lower fusing bodies, 2,250 or 2,300 degrees, will answer every purpose, and I believe experiments have shown them just as strong as high fusing bodies, I doubt if White's body has any lead in it at all, because it has a beautiful clear and transluscent appearance, and it seems to blend harmoniously with the high fusing body as much as any of the others.

I have not used bands on crowns in the anterior teeth for several years, and I do not really see where a half band is needed on the bicuspid crowns, if the root is properly shaped. I think you get just as strong a crown in porcelain as if banded. I do not see any advantage in allowing the platinum to extend over the edge of the root. I think if we allow the porcelain to come directly in contact with the root and absolutely flush with it the gum tissue will come over that better and it is subject to less irritation. Some might say that the contact of the porcelain against the end of the root might cause it to break. I do not see how it could if it properly fits the root and is properly cemented into place.

I do not think there is as much porcelain used as there should be now. I believe that too many porcelain furnaces are put on the back shelf, and some say they can not afford to buy them. I believe this is largely due to following improper principles, and also to those who have tried it, thinking it was too simple a process, and after seeing done some beautiful piece of work at a clinic, some men have gone to their office thinking they could go to work and get the same results, forgetting how much time has been put into learning to make such beautiful fillings.

I still stick to the statement I once made, and which Dr. Thompson repeated to-night, that I think the application of porcelain is going to be limited by the ability of the operator only, but I have modified my use of it somewhat, whereas I did use porcelain in molars cavities, because I considered it from a hygienic point the best thing to have, it is not always as good as it might be. I still use it in nearly all bicuspids, and the edges don't break away if the walls of the cavity are properly prepared, but I think the gold inlay has properly taken its place in the molars.

Dr. C. H. Oakman: I enjoyed the very excellent paper of Dr. Lyons, and would say that it showed a good deal of original thought. I could not help but feel pleased at his suggestion that after the matrix was prepared, you should obtain all the retention possible in the cavity. Dr. Reeves, you will remember, tabooed the idea of any retaining form, but advocated cavities on the principal of a saucer-shaped preparation, if you please, no undercuts whatever, these were not necessary. Our fillings came out, quite to our displeasure, frequently. We met one another on the street and asked: "Are you doing much porcelain now?" Answer, "Yes, doing lots of it."

Question: "Fillings sticking?"

Answer: "No."

Of course we had to own up to our friends—we did not tell our patients that. Then by etching the inlay, our fillings began to stay better. Then we thought we would take a chance and get retention in the cavity by the use of small fine burs, and they stayed still better, and when Dr. Lyons spoke of that it pleased me very much, because I really think that it is along the right line, and I know that every man and woman here that is doing porcelain work, if he follows that principal, he is getting better results than ever before. How many times would our inlays come out with very little cement adherent to the inlay, because the part of the inlay fitting against the tooth was as smooth as the proximal facing of the inlay. I think the acid etching has been a saving factor.

Dr. Thompson spoke of a point which I would like to emphasize, in regard to using a very fine platinum to burnish over the roots. I know Dr. Thompson has done that for a number of years with excellent results, and I have made a number of crowns with a half band or two-thirds band, making the band in the usual way and cutting it out afterwards and bringing the cap up to the level of the root; after the post was in place, took it to the furnace and put it on porcelain and brought it back, finding that it did not fit and so had to take it off, it was necessary to burnish the platinum over the root again. By using a very fine platinum about a thousand fine, the manipulation is necessarily very exacting, but the results obtained are of the best, I think.

Porcelain for molars I never use; however, I have seen some very excellent work done with it, but I have always fought shy of it for the mere reason that I do not think it is strong enough. I have seen a number of them broken in the mouth, but that is not the only reason. If the crown is on the anterior teeth, frequently the labial aspect of the tooth is broken off because there is only a slight amount of porcelain covering the band, and it is a frequent occurrence with many, I think, that the porcelain breaks at the point of the thinnest margin.

I notice our friend Dr. Thompson has spoken of a double pin going into both roots of the bicuspid. I think he has success with them, but I have never used it and stuck to the one pin. I want to thank Dr. Lyons for coming here and giving us this excellent paper to-night.

Dr. F. W. MacDonald: I was thinking, as I sat here, how subdued we all were when discussed the porcelain of today; we are not as bombastic in our utterances as we were five or six years ago.

I am glad Dr. Raymond modified that statement of his;

I am sorry Dr. Thompson could not do so also in regard to the use of porcelain, as depending altogether on his ability to use it as to where he should use it. You could use almost any other substance in the same way. What we strive to do is to produce an efficient filling as well as an aesthetic one, and I have yet to see porcelain inlays where the margin is not properly prepared that have stood the test of time in the occluded surface molars and bicuspids. I think Dr. Lyons could not have struck a happier medium in his paper all the way through. He was not an extremist, and I believe he has put porcelain in its proper place in practice.

There is one method in which I use porcelain a great deal and in which I think a great many of us could use it to good advantage, and that is in connection with our Davis crowns. It is very difficult sometimes to get crowns that are really the proper shape or the right shade—they might be just a little bit too narrow, although a very good shade. I very frequently use porcelain after I have fitted a crown somewhat, and modify the shade and change the shape; we can do that very easily, at the latter part of the operation, and can produce some very artistic results. Many dentists, especially the younger men, who are not enthusiastic in regard to inlays, claim that they do not get the fees that remunerate them, but I claim that if you use porcelain in no other operation than for that one thing, you would find that porcelain would pay in any practice.

I would liked to have heard a little more discussion in regard to the staining of margins than we have heard to-night. Of course I can well realize that Dr. Lyons in his paper has had a great deal of trouble to furnish so many little details in regard to the making of inlays and making porcelain crowns, that he had not the time to dwell on these little matters, but the margin is really the thing that has concerned me most of all in many inlays. If we could get inlays to remain in place, we could construct them to look nice for a couple or three years, and the margin would begin to show itself, although the margin at the beginning looked very well; it was not altogether invisible, and yet certain patients have certain stains in the mouth that we simply cannot get rid of, and these stains will penetrate the slightest crevice and these slight crevices around our porcelain inlays after, say two or three years, certainly will show stains, and it has been my greatest bane in porcelain work to deal with those stains. I have not been able to do it successfully. I have tried in many ways, but they always come back. I do not find that in every case, but in enough cases to make that feature of porcelain work a very trying one.

I agree with Dr. Thompson's remarks that we have had a very good discussion of crown work, and the men who have kept up fees and care to do that class of work, I think it is the most artistic work that the operator can do. He can get every desirable result almost; and when Dr. Thompson speaks about the strength of the crown built up as he built it up, I believe it. I have a porcelain crown in my own mouth that I have given excessive use, due to the fact that one on the other side of my mouth has been disarranged for some time, and it has stood the test in every way. Not only that, but as Dr. Lyons has said, the hygienic feature of procelain work in the posterior side of the mouth is certainly good. The trouble is with a great many men who have done porcelain work in the past, they made some statements that our own practice could not back up, and we sometimes felt that we did not have the ability to do this work, but after seeing the work of a great many men whom we think have this ability, we know that they must mean it or they would not make such statements, and keep at the work.

DR. J. M. THOMPSON: I was told once that a jest had no place in business matters. From the remarks of Dr. MacDonald I see that I have been misunderstood. I said that the old assertion that the use of porcelain was limited by the use of the operator, I still believe. Then I said, "Think it Over." Now I have been called a sort of a dry joker in many instances, and that is one of my dry jokes. I do not want to have the impression to arise among the gentlemen here to-night that I am setting up myself before any other individual, as the greatest good in the line of porcelain work, because I do not believe there is any man in the profession of dentistry to-day who can put porcelain in every place and make it stand on stress. I am putting in just as many gold inlays and am just as enthusiastic in every way over gold inlays as I am over porcelain, and I think I have just as many failures in connection with porcelain work as the average man, and if some of you have had the privilege of correcting them and putting in gold inlays in their stead, that is your gain, and I hope you will not misunderstand me in my little remark that I made, that I still believe that it applied everywhere, because I do not.

Dr Lyons: I certainly want to express my deep appreciation for the kind manner in which you have received my paper to-night. I appreciate it very much indeed. I do not know that I can add anything to what has already been said. I am very glad indeed that Dr. Thompson has squared himself, because I firmly believe that it was statements of that kind which were made by eminent men a few years ago that has done a great deal to discredit porcelain. Men took up porcelain work believing those statements, and placed porcelain where it was not indicated and where it did not belong, and failed, and then, instead of placing the blame where it belonged they condemned the material. I firmly believe that porcelain has its place, and I also believe that the dentist's equipment is not complete unless he has a complete porcelain outfit, because there are places where porcelain is indicated and nothing else will take its place, that we have at the present time.

I was glad to hear Dr. Thompson bring out the fact of his using the Davis crown. I believe by its use and the Consolidated body we can produce results that we cannot get with any other method.

The point that Dr. Raymond emphasized in regard to the difference in the glaze of the natural teeth is well taken, and before beginning the operation for the porcelain inlay

I believe we should study the glaze of that particular case, and then try to reproduce the glaze of the natural teeth on the inlay. Sometimes I have found that I could get better results in color by dulling the glaze a little by the cuttle fish disc, where the glaze of the natural teeth was dull. In order to get the proper colors and the proper refraction of light you have got to have the reflection on inlay exactly the same as on that of the natural tooth. Your colors may be all right, but if your reflection is not the same, the appearance will be changed.

In regard to the comment that Dr. MacDonald made as to staining of the margins, I have found that if you are particular in etching your inlay not to etch the margins, that the adaptation will be a little bit better. Cover the margins of the inlay with wax so as to prevent their etching. Your adaptation will be closer and there is not much tendency for the margins to take up stains.

Dr. G. C. Bowles: I regret that the contraction and expansion of porcelain has not been considered by the essayist. Dr. Clarence Graves, in the March *Cosmos*, had an admirable paper showing it was impossible to fuse porcelain around platinum without having an area of fractured porcelain, and several in speaking of Dr. Thompson's Davis crown and his thin platinum piece have said that they made a strong crown. It is strong because the expansion and contraction of the porcelain has not taken place to fracture the porcelain. Porcelain expands six points to platinum's fourteen. When the platinum expands, the porcelain is fused at the extreme points; as it is re-heated over and over again, the edges not only thin and contract, but become more friable. In this paper, Dr. Graves says that of two facings in the same mouth, one having pins and one not having them, the one not having the pins is three times as strong as the one having them. After heating them to fusing degree he found that those not having pins when heated were three times as strong as those having pins. Every one doing porcelain work should carefully study the paper of Dr. Graves. I

notice how the commercial houses take hold of these facts. Within a day or so I received a catalogue from the Twentieth Century Tooth people and note that they embody this principle in their advertisement.

DR. BECKER: Since last spring I have used the Davis crown and cast inlay method of setting them to the root, especially on bicuspids. I like two pins there, and find that the wax does not only come down and fill the space alongside of the pin and fill the hollow in the root perfectly, but also comes up into the tooth around the pin that the Davis people furnish you, and takes it up so that there is very little cement needed and little grinding. A person certainly can afford to set these crowns for remunerative fees.

DR. OAKMAN: A point occurred to me when Dr. MacDonald spoke about the margin. I wonder if every one boils out the acid from the inlay after etching it. I do not know but this might be a factor, or its porosity may have something to do with its discoloration.

